# Optimization of Ultrasound-Assisted Extraction of Natural Antioxidants from Sugar Apple (*Annona squamosa* L.) Peel Using Response Surface Methodology

**DOI:** 10.3390/molecules201119708

**Published:** 2015-11-17

**Authors:** Gui-Fang Deng, Dong-Ping Xu, Sha Li, Hua-Bin Li

**Affiliations:** 1 Department of Clinical Nutrition, Nanshan Hospital, Shenzhen 518052, China; 2 Guangdong Provincial Key Laboratory of Food, Nutrition and Health, School of Public Health, Sun Yat-Sen University, Guangzhou 510080, China; xudongping1989@163.com (D.-P.X.); lishasl0308@163.com (S.L.)

**Keywords:** sugar apple peel, antioxidant, ultrasound-assisted extraction, response surface methodology

## Abstract

Sugar apple (*Annona squamosa* L.) is a popular tropical fruit and its peel is a municipal waste. An ultrasound-assisted extraction method was developed for the recovery of natural antioxidants from sugar apple peel. Central composite design was used to optimize solvent concentration (13.2%–46.8%), ultrasonic time (33.2–66.8 min), and temperature (43.2–76.8 °C) for the recovery of natural antioxidants from sugar apple peel. The second-order polynomial models demonstrated a good fit of the quadratic models with the experimental results in respect to total phenolic content (TPC, *R*^2^ = 0.9524, *p* < 0.0001), FRAP (*R*^2^ = 0.9743, *p* < 0.0001), and TEAC (*R*^2^ = 0.9610, *p* < 0.0001) values. The optimal extraction conditions were 20:1 (mL/g) of solvent-to-solid ratio, 32.68% acetone, and 67.23 °C for 42.54 min under ultrasonic irradiation. Under these conditions, the maximal yield of total phenolic content was 26.81 (mg GA/g FW). The experimental results obtained under optimal conditions agreed well with the predicted results. The application of ultrasound markedly decreased extraction time and improved the extraction efficiency, compared with the conventional methods.

## 1. Introduction

Fruit wastes are one of the main sources of municipal wastes. Due to the high consumption and industrial processing of fruit edible parts, fruits residues (principal peels and seeds) are generated in large quantities in large cities and become a severe environmental issue. However, if the phytochemicals of fruit residues were extracted effectively by applying efficient extraction technologies, their value could be added [1,2]. Recently, the contents of natural antioxidants were found to be very high in the peel and seed of some fruits [3,4,5,6]. Therefore, it would be beneficial if the fruit residues could be used to recover natural antioxidants especially phenolic compounds, making them fully used in the food, pharmaceutical, as well as cosmetics industry [7].

*Annona squamosal* L. (Annonaceae) is an important plant and widely distributed in tropical America and Asia [8]. It has long been used as a folk medicine for the treatment of epilepsy, dys entry, cardiac problem, worm infection, constipation, hemorrhage, bacterial infection, dysuria, fever, and ulcers [9]. *Annona squamosal* bears edible fruits called sugar apple. It is a popular tropical fruit, typically globular or heart-shaped, 5–10 cm in diameter, with many round protuberances. The pulp is sweetly aromatic with a custard-like flavor, and is widely used to prepare juices, jellies, and compotes. The processing of the fruits will leave behind a substantial amount of non-edible parts. In previous studies, extremely high phenolic content and antioxidant capacities were found in sugar apple peel, indicating its potential to be utilized as a resource of natural antioxidants [4,8]. So far, there were few studies reporting the optimization extraction of natural antioxidants from sugar apple peel. Recently, ultrasound-assisted extraction (UAE) has been successfully used for the extraction of bioactive compounds from plant matrix [10,11,12,13]. It is a simple, efficient, and inexpensive alternative to conventional extraction techniques [14]. Ultrasound has a mechanical effect that enables greater penetration of the solvents into the matrix and increases the surface contact between the solid and the liquid [12]. Ultrasonic cavitation creates shear forces that break cell walls mechanically and enhance mass transfer. Therefore, ultrasound-assisted extraction leads to the improvement of the extraction efficiency [15,16].

The extraction efficiency and antioxidant capacity of natural antioxidants could be markedly influenced by many factors, such as solvent type and concentration, solvent-to-solid, as well as extraction temperature and time [10]. Therefore, it is necessary to optimize the extraction conditions to obtain optimal antioxidant recovery. Response surface methodology (RSM) is an often-used optimization technique that applies sequential experimental techniques to survey a domain of interest, focusing on the most important variables and their effects, as well as the interactions between them, to build an empirical model [17,18]. RSM has been successfully used in optimizing the extraction conditions of phenolic compounds from food matrix [10,11,19,20,21,22]. Therefore, in the present study, the extraction conditions of natural antioxidants from sugar apple peel was optimized by applying the RSM, employing a three-variable, five-level central composite design, evaluating the influence of solvent concentration, ultrasonic time, and temperature in the process of extraction. The agreement of the experimental results obtained under optimal conditions to the predicted results demonstrated the good suitability of the fitted model. The application of ultrasound markedly decreased extraction time, and improved the extraction efficiency compared with the conventional methods.

## 2. Results and Discussion

### 2.1. Preliminary Results

**Figure 1:**
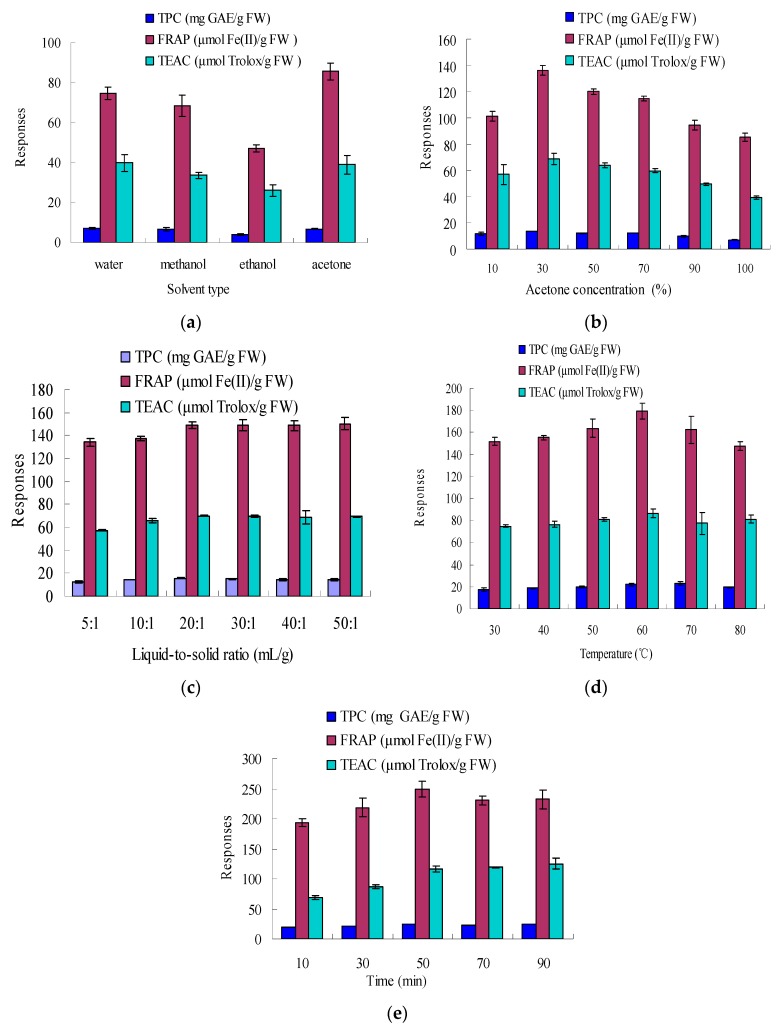
Effects of solvent type, solvent-to-solid ratio, acetone concentration, ultrasonic time, and temperature on the responses. Extraction conditions: (**a**) 10:1 mL/g, 25 °C, 30 min; (**b**) 10:1 mL/g, 25 °C and 30 min; (**c**) 30% acetone, 25 °C, 30 min; (**d**) 30% acetone, 20:1 mL/g, 30 min; and (**e**) 30% acetone, 20:1 mL/g, 60 °C.

In the preliminary experiment of our study, the effects of solvent type, solvent-to-solid ratio, acetone concentration, ultrasonic time, and temperature on the extraction of natural antioxidants from sugar apple peel were studied. Under the same extraction conditions (10:1 mL/g, 25 °C and 30 min), the responses followed a similar trend among all the solvents tested (Figure 1a).

In the literature, several frequently-used solvents were reported, such as ethanol, methanol, and acetone [12,17,18,19,20,21]. In our study, compared to ethanol and methanol, acetone yielded higher responses and was more effective in the extraction of antioxidants from sugar apple peel (Figure 1a). In addition, the extraction efficiency using water was also good. The polarity of the solvent could play an important role in the extraction efficiency [13]. Therefore, the effect of acetone-water at different ratios as extracting solvents was investigated further, and 30% acetone was optimal (Figure 1b). As for the effect of the solvent-to-solid ratio, six ratios (5:1, 10:1, 20:1, 30:1, 40:1, 50:1 mL/g) were investigated over a 30 min ultrasonic period, with 30% acetone, at 25 °C. The results were displayed in Figure 1c. From 10:1 to 20:1, the responses were markedly increased (*p* < 0.05). At the solvent-to-solid ratio of 20:1, a plateau in the mass transfer was reached. There were, indeed, no significant differences for solvent-to-solid ratios between 30:1, 40:1, 50:1, and 20:1 (*p* > 0.05). In consideration of the solvent consumption and extraction efficiency, solvent-solid ratio of 20:1 (mL/g) was selected. The effects of extraction temperature and time were shown in Figure 1d,e, and the optimal values were 60 °C and 50 min, respectively. According to the results shown in Figure 1b–e, the effects of acetone concentration, ultrasonic temperature, and time were more significant on the extract efficiency than solvent-solid ratio; thus, they were further investigated as independent processing variables by RSM.

### 2.2. Fitting the Models

The results of 20 runs using CCD design were shown in Table 1, which included the design and observed responses TPC, FRAP, and TEAC. Total phenolic contents ranged from 15.93 to 26.97 mg GAE/g FW. FRAP and TEAC assays were applied to determine the antioxidant capacities of the extracts. The results showed that FRAP and TEAC values varied from 152.89 to 271.93 µmol Fe(II)/g FW, and 72.49 to 124.84 µmol Trolox/g FW, respectively.

**Table 1:** Central composite design arrangement in terms of the independent variables (*X*_1_, *X*_2_, and *X*_3_) and their observed responses, including Total Phenol Contents (TPC), Ferric-Reducing Antioxidant Power(FRAP), and Trolox Equivalent Antioxidant Capacity (TEAC).

Run	*X*_1_	*X*_2_	*X*_3_	TPC (mg GAE/g FW)	FRAP (μmol Fe(II)/g FW)	TEAC (μmol Trolox/g FW)
1	40 (1)	60 (1)	50 (−1)	21.55	232.39	81.89
2	30 (0)	50 (0)	60 (0)	26.24	255.54	121.03
3	20 (−1)	40 (−1)	50 (−1)	22.44	180.86	73.71
4	30 (0)	50 (0)	76.8 (1.68)	26.37	259.93	100.09
5	20 (−1)	40 (−1)	70 (1)	20.72	257.57	85.88
6	30 (0)	50 (0)	60 (0)	24.88	252.64	115.63
7	40 (1)	40 (−1)	70 (1)	23.75	258.96	113.88
8	30 (0)	50 (0)	60 (0)	26.18	252.32	118.12
9	40 (1)	40 (−1)	50 (−1)	19.27	200.68	93.77
10	30 (0)	66.8 (1.68)	60 (0)	26.91	252.11	97.59
11	46.8 (1.68)	50 (0)	60 (0)	15.96	207.75	102.42
12	40 (1)	60 (1)	70 (1)	24.44	234.43	95.68
13	20 (−1)	60 (1)	70 (1)	23.51	209.25	90.78
14	30 (0)	50 (0)	60 (0)	26.94	264.86	124.86
15	30 (0)	33.2 (−1.68)	60 (0)	23.01	271.93	113.64
16	20 (−1)	60 (1)	50 (−1)	24.72	179.89	93.36
17	30 (0)	50 (0)	60 (0)	25.84	247.82	122.45
18	30 (0)	50 (0)	60 (0)	26.97	251.07	113.72
19	13.2 (−1.68)	50 (0)	60 (0)	15.93	157.39	72.49
20	30 (0)	50 (0)	43.2 (−1.68)	21.87	152.89	77.15

The analysis of variance (ANOVA) of the quadratic regression models for TPC, FRAP, and TEAC showed that the models were significant (*p* < 0.0001) with *F*-values of 22.23, 42.05, and 27.36, respectively (Table 2). The coefficient of determination (*R*^2^) is a measure of degree of fit [23]. For a good fit of a model, *R*^2^ should be at least 0.80 [24]. In this study, the *R*^2^ values of the second-order polynomial models for TPC, FRAP, and TEAC were 0.9524, 0.9743, and 0.9610, respectively, which implied that 95.24%, 97.43%, and 96.10% of the variations could be explained by the fitted quadratic models, respectively. Furthermore, the absence of any lack of fit (*p* > 0.05) also strengthened the reliability of all models. The models were used for the construction of three dimensional response surface plots to predict the relationship between the responses and independent variables.

**Table 2:** Analysis of variance (ANOVA) of the quadratic model and lack of fit.

Response	Source	Sum of Squares	Mean Squares	DF	*F*-Test	*p*-Value
TPC	Model	205.27	22.81	9	22.23	<0.0001 ^a^
	Lack of Fit	7.25	1.45	5	2.41	0.1784 ^b^
	Residual	10.26	1.03	10		
	Pure Error	3.01	0.6	5		
	*R*^2^	0.9524				
FRAP	Model	25,638.96	2848.77	9	42.05	<0.0001 ^a^
	Lack of Fit	505.72	101.14	5	2.94	0.1305 ^b^
	Residual	677.47	67.75	10		
	Pure Error	171.75	34.35	5		
	*R*^2^	0.9743				
TEAC	Model	5185.6	576.18	9	27.36	<0.0001 ^a^
	Lack of Fit	120.8	24.16	5	1.34	0.3765 ^b^
	Residual	210.62	21.06	10		
	Pure Error	89.83	17.97	5		
	*R*^2^	0.9610				

Note: ^a^ Significant at 5.0% (*p* < 0.05); ^b^ Non-significant.

### 2.3. Effects of Independent Variables on Total Phenolic Contents

Total phenolic contents of sugar apple peel extracts obtained by ultrasound-assisted extraction were shown in Table 1. The experimental data was subjected to a multiple regression analysis, and the model coefficients were evaluated for significance (Table 3). Regression equation (Equation (1)) showed the relationship between acetone concentration, time, and temperature for the extraction of total phenolic compounds.

(1) *Y*_TPC_ = *2*6.14 − 0.17*X*_1_ + 1.07*X*_2_ + 0.88*X*_3_ − 0.26*X*_1_*X*_2_ + 1.29*X*_1_*X*_3_ − 0.13*X*_2_*X*_3_ − *33.38X*_1_^2^ − 0.19*X*_2_^2^ − 0.48*X*_3_^2^

**Figure 2:**
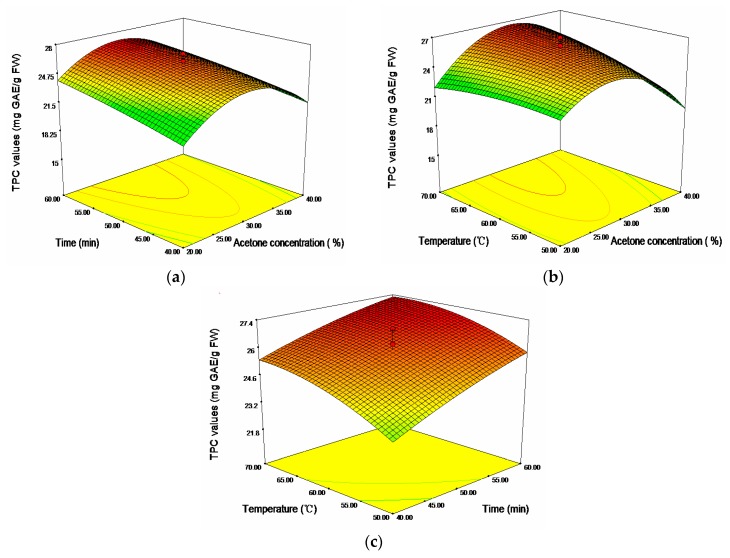
Response surface plots for the effects of (**a**) acetone concentration/time; (**b**) acetone concentration/temperature; and (**c**) time/temperature on the total phenolic content (TPC).

It was evident that the linear effects of time (*X*_2_) and temperature (*X*_3_), and the cross product effect between acetone concentration and temperature (*X*_1_, *X*_3_), were positive and significant at *p* < 0.01, while the quadratic effect of acetone concentration (*X*_1_^2^) were negative and significant at *p* < 0.001. Figure 2 showed the 3D response surfaces plots of TPC of sugar apple peel extracts. Seen from Figure 2b, TPC increased with the increase of ultrasonic temperature up to about 67.23 °C, and then began to decline with further rise of temperature. A rise in ultrasonic temperature increased the cavitational intensity, promoting the disruption of the plant tissue and cell wall, and enhancing hydrolysis of the bonds of bound phenolic compounds as well as phenolics solubility [13,25]. At higher temperature, solvent viscosity and surface tension were reduced, which could also lead to a better extraction of phenolic compounds [26]. However, some of the thermolabile phenolic compounds might be degraded after the optimum ultrasonic temperature was reached, thereby leading to a decline phenolic content. An extended extraction time favored the extraction of phenolic compounds. However, acetone concentration exerted a quadratic effect on the response; hence TPC gradually mounted up with the increase of acetone concentration, and achieved optimum values at about 32.68%, followed by a decline of TPC with further increase of acetone concentration.

**Table 3:** Estimated coefficients of the fitted second-order polynomial for the investigated responses and their signification based on analysis of variance (ANOVA).

Model Parameter	Coefficient	TPC	FRAP	TEAC
Intercept	β_0_	26.14	253.89	119.34
Linear				
*x*_1_	β_1_	−0.17	13.45 ^a^	6.73 ^a^
*x*_2_	β_2_	1.07 ^c^	−5.53 ^b^	−2.38
*x*_3_	β_3_	0.88 ^c^	25.37 ^a^	6.01 ^a^
Quadratic				
*X*_1_^2^	β_11_	−3.38 ^a^	−24.35 ^a^	−14.91 ^a^
*X*_2_^2^	β_22_	−0.19	3.8	−5.1 ^c^
*X*_3_^2^	β_33_	−0.48	−15.91 ^a^	−11.12 ^a^
Cross product				
*X*_1_ × *X*_2_	β_12_	−0.26	7.06 ^b^	−6.83 ^c^
*X*_1_ × *X*_3_	β_13_	1.29 ^c^	−5.72	3.04
*X*_2_ × *X*_3_	β_23_	−0.13	−12.95 ^c^	−2.63

Note: ^a^ Significant at 0.1% (*p* < 0.001); ^b^ Significant at 5% (*p* < 0.05); ^c^ Significant at 1% (*p* < 0.01).

### 2.4. Effects of Independent Variables on the Antioxidant Capacities

The experimental results of antioxidant capacities of sugar apple peel extracts measured by FRAP and TEAC assays were performed regression analysis and the results were shown in Table 3. The relationship of antioxidant capacities between the three independent variables was present in the regression equation (Equations (2) and (3)):

(2) *Y*_FRAP_ = 253.89 + 13.45*X*_1_ − 5.53*X*_2_ + 25.37*X*_3_ + 7.06*X*_1_*X*_2_ − 5.72*X*_1_*X*_3_ − 12.95*X*_2_*X*_3_ − 24.35*X*_1_^2^ + 3.8*X*_2_^2^ − 15.91*X_3_*^2^

(3) *Y*_TEAC_ = 119.34 + 6.73*X*_1_ − 2.38*X*_2_ + 6.01*X*_3_ − 6.83*X*_1_*X*_2_ + 3.04X_1_X_3_ − 2.63*X*_2_*X*_3_ − 14.91*X*_1_^2^ − 5.1*X*_2_^2^ − 11.12*X_3_*^2^

**Figure 3:**
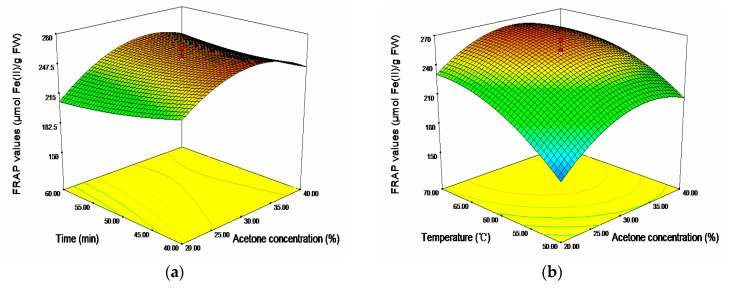

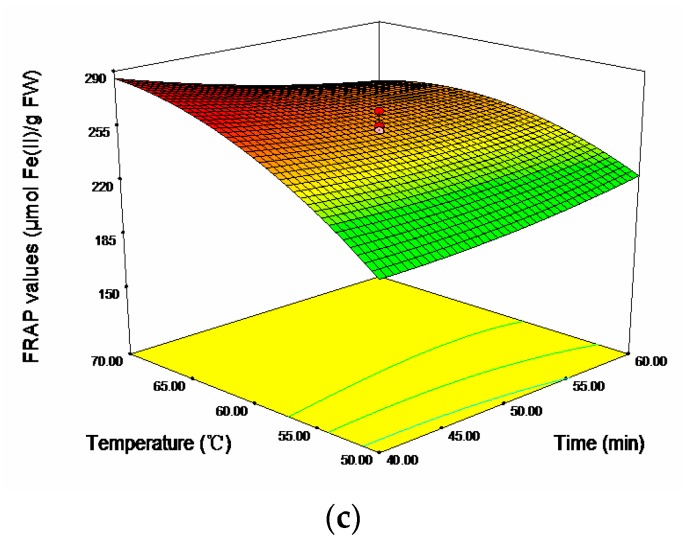
Response surface plots for the effects of (**a**) acetone concentration/time; (**b**) acetone concentration/temperature; and (**c**) time/temperature on the FRAP values.

In term of FRAP, acetone concentration (*X*_1_) and temperature (*X*_3_) showed significantly positive linear effects and negative quadratic effects, respectively (*p* < 0.001). Ultrasonic time showed significantly negative linear effect on the response (*p* < 0.05). The cross product effect between acetone concentration (*X*_1_) and time (*X*_2_) was positive and significant at *p* < 0.05, whereas the cross product effect between time (*X*_2_) and temperature (*X*_3_) was negative and significant at *p* < 0.01. The 3D response surfaces plot for the effects of independent variables (*X*_1_, *X*_2_, and *X*_3_) on FRAP values were given in Figure 3. As shown, the region of 30%–35% acetone concentration, 60–70 °C and 40–45 min would achieve a higher FRAC values (maximum 276.47 μmol Fe(II)/g FW).

**Figure 4:**
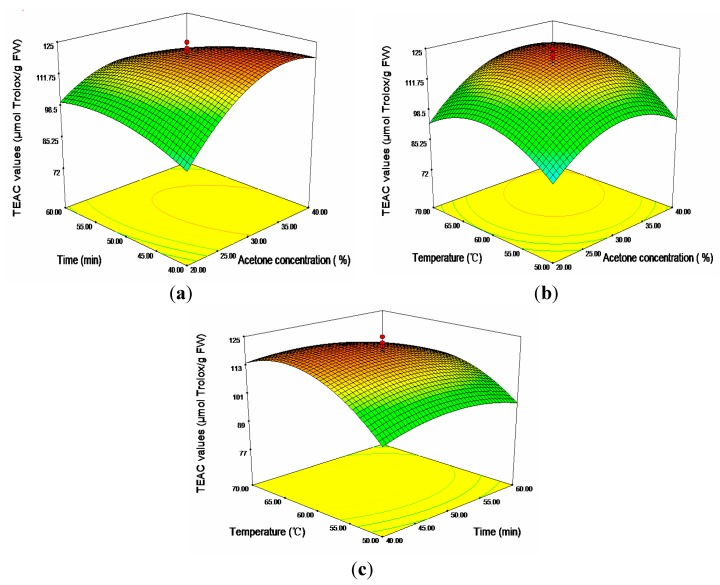
Response surface plots for the effects of (**a**) acetone concentration/time; (**b**) acetone concentration/temperature; and (**c**) time/temperature on the TEAC values.

In aspect of TEAC, as shown in Table 3, except linear term of time (*X*_2_), cross product terms of time (*X*_2_) and temperature (*X*_3_), all the terms had significant effects on the antioxidant capacities (*p* < 0.01). The 3D response surfaces plot Figure 4 showed the relationship between the TEAC values and the three independent variables (*X*_1_, *X*_2_, *X*_3_). Seen from Figure 4c, when the temperature rose from 50 to 65 °C, an apparent increase of TEAC values could be found. However, further increase was limited when the temperature rose from 65 to 70 °C. It was evident that acetone concentration exhibited a similar effect on TEAC values as temperature. An apparent increase of TEAC values was recorded from 20%–32.68% (maximum 121.16 μmol Trolox/g FW), followed by a moderate decline of TEAC values with further increase of acetone concentration.

### 2.5. Verification of Results

The optimum extraction conditions obtained by RSM was used to validate the predictive model of extraction for TPC, FRAP, and TEAC values from sugar apple peel. Seen from Table 4, extraction in 32.68% acetone at 20:1 (mL/g), 67.23 °C for 42.54 min under ultrasonic irradiation could result in optimal TPC (26.81 mg GA/g FW), FRAP (280.36 μmol Fe(II)/g FW), and TEAC (120.95 μmol Trolox/g FW) values from sugar apple peel. The predicted values matched well with the experimental values obtained using optimum extraction conditions, which validated the suitability of the fitted RSM model. Furthermore, compared with conventional methods such as Soxhlet extraction and maceration extraction (Table 4), the application of ultrasonic system for the extraction of antioxidants markedly decreased extraction time, and improved the extraction efficiency, as shown by the 27.7%–43% and 29.7%–35% increase of extraction rate were achieved, respectively.

**Table 4:** Comparison of experimental results with predicted results obtained by the fitted model and the results obtained by the conventional method.

Response Variables	Extraction Methods			
	Predicted	Experimental *	Soxhlet **	Maceration ***
TPC (mg GA/g FW)	25.70	26.81	19.59	20.67
FRAP (μmol Fe(II)/g FW)	276.47	280.36	196.10	211.39
TEAC (μmol Trolox/g FW)	121.16	120.95	94.69	89.62

Note: *: time: 42.54 min; temperature: 67.23 °C; **: time: 4 h; temperature: 100 °C; ***: time: 24 h; temperature: 37 °C. Other conditions of the three methods were 32.68% acetone and 20:1 (mL/g) of solvent-to-solid ratio.

## 3. Experimental Section

### 3.1. Chemical Reagents and Samples

The compounds 6-hydroxy-2,5,7,8-tetramethylchromane-2-carboxylic acid (Trolox), 2,4,6-tri(2-pyridyl)-S-triazine (TPTZ), 2,2′-azinobis(3-ethylbenothiazoline-6-sulfonic acid) diammonium salt (ABTS), Folin–Ciocalteu’s phenol reagent and gallic acid were purchased from Sigma–Aldrich (St. Louis, MO, USA). Acetone, ethanol, methanol, acetic acid, hydrochloric acid, potassium persulphate, iron(III) chloride hexahydrate, iron(II) sulfate heptahydrate, sodium acetate, and sodium carbonate were of analytical grade and obtained from Tianjin Chemical Factory (Tianjin, China). Deionized water was used throughout the experiment.

Sugar apples were collected from markets in Guangzhou, China. Samples were cleaned with deionized water and the peel was separated. Immediately, the separated peel was ground into fine particles with a special grinder.

### 3.2. Extraction Procedure

The ultrasound-assisted extraction process was performed in a ultrasonic device (KQ-600E, 40 kHz, Changzhou Nuoji Instrument Company, Changzhou, China) with an electric power of 600 W (*i.e.*, acoustic power of 0.38 W/cm^2^), heating power of 800 W, equipped with a digital timer and a temperature controller. In RSM test, sample particles (0.5 g) were sonicated in the acetone (10 mL) for different time at set temperature. After sonication, the sample was centrifuged at 9600 g for 20 min, and then the supernatant was collected and diluted for analysis. The reaction process happened in a 15 mL centrifuge tube with screw cap and conical bottom, which was fixed in a plastic rack placing in the ultrasonic device.

For comparison, in Soxhlet method, 7.5 g sample was extracted using 150 mL of 32.68% acetone, at 100 °C for 4 h. In maceration method, 0.5 g sample was extracted with 10 mL of 32.68% acetone in a shaking water bath (100 rpm) at 37 °C for 24 h.

### 3.3. Ferric-Reducing Antioxidant Power (FRAP) Assay

The FRAP assay was performed according to the procedure described by Benzie and Strain [27] with minor modifications. In this assay, 100 μL of the diluted sample was added to 3 mL of the FRAP reagent and the reaction was monitored after 4 min at 593 nm. The results were expressed as µmol Fe(II)/g fresh weight (FW) of fruit peel.

### 3.4. Trolox Equivalent Antioxidant Capacity (TEAC) Assay

The TEAC assay was performed based on the protocol established previously [28] with minor modifications. Briefly, the ABTS^•+^ stock solution was prepared from 7 mmol/L ABTS and 2.45 mmol/L potassium persulfate in a volume ratio of 1:1, and then incubated in the dark at room temperature for 16 h and used within two days. A 100 μL of the tested sample was mixed with 3.8 mL ABTS^•+^ working solution and the absorbance was detected at 734 nm after incubation at room temperature for 6 min. The percent of inhibition of absorbance at 734 nm was calculated and the results were expressed as µmol Trolox/g FW of fruit peel.

### 3.5. Determination of Total Phenolic Content

Total phenolic contents were determined with Folin–Ciocalteu method [29]. Briefly, 0.50 mL extract was mixed with 2.5 mL of 1:10 diluted Folin–Ciocalteu reagent. After 4 min, 2 mL of saturated sodium carbonate solution was added. The mixture was incubated in dark at room temperature for 2 h and its absorbance was detected at 760 nm. Gallic acid was used for calibration, and the results were expressed as mg of gallic acid equivalent (mg GAE) per 100 g FW of fruit peel.

### 3.6. Experimental Design

Optimization of extraction conditions for natural antioxidants from sugar apple peel were carried out using response surface methodology (RSM). A three-variable, five level central composite design (CCD) was applied to determine the best combination of extraction variables. Acetone concentration (*X*_1_), ultrasonic time (*X*_2_), and ultrasonic temperature (*X*_3_) were chosen for independent processing variables. The range and center point values of three independent variables presented in Table 5 were based on the results of preliminary experiments in our laboratory. The CCD in the experimental design consists of twenty factorial points including six replicates of the central point (Table 1), and the experiment was carried out in a standard order. TPC, FRAP, and TEAC were selected as the responses for the combination of the independent variables. All the experiments were performed in quadruplicate, and the average values of the responses were reported. The experimental data from the CCD was analyzed using response surface regression and fitted to a second-order polynomial model (Equation (4)):

(4) $$Y\;=\;\beta_{i}\;+\;{\Sigma \beta }_{i}X_{i}\;+\;{\Sigma \beta }_{ii}X_{i}^{2}\;+\;{\Sigma \beta }_{ij}X_{i}X_{j}$$

*Y* is the predicted response, β_0_ is an intercept, β_*i*_, β_*ii*_, and β_*ij*_ are the coefficients of the linear, quadratic, and interaction terms, respectively. *X_i_* and *X_j_* are coded independent variables.

**Table 5:** Coded and uncoded levels of the independent variables.

Independent Variables	Code Units	Coded Levels				
		−1.68	−1	0	1	1.68
Acetone concentration (%, *v*/*v*)	*X*_1_	13.2	20	30	40	46.8
Ultrasonic time (min)	*X*_2_	33.2	40	50	60	66.8
Ultrasonic temperature (°C)	*X*_3_	43.2	50	60	70	76.8

The regression and graphical analysis of the experimental data were performed by Design Expert version 7.1.3 software (Stat-Ease Inc., Minneapolis, MN, USA). The suitability of the model was checked by evaluating the lack of fit, coefficient of determination (*R*^2^), and the Fisher test value (*F*-value) generated from the analysis of variance (ANOVA) by the software. The difference was considered significant at *p* < 0.05.

### 3.7. Verification of Model

The optimum extraction conditions for natural antioxidants depended on acetone concentration, ultrasonic time, and temperature, and were obtained using RSM. For verification of the model, extraction of antioxidants from sugar apple peel was performed under optimal conditions and the extracts were determined. The experimental and predicted values were compared to check the validity of the model.

## 4. Conclusions

An ultrasound-assisted extraction method has been developed for the recovery of natural antioxidants from sugar apple peel. Response surface methodology was used to optimize the experimental variables such as solvent concentration, ultrasonic time, and temperature. The *R*^2^ values of the second-order polynomial models for TPC, FRAP, and TEAC were 0.9524, 0.9743, and 0.9610, respectively, which indicated a good fit to the quadratic models. The optimal extraction conditions were 20:1 (mL/g) of solvent-to-solid ratio, 32.68% acetone and 67.23 °C for 42.54 min under ultrasonic irradiation. The application of ultrasound markedly decreased extraction time, and improved the extraction efficiency compared with the conventional methods.
